# Draft Genome Sequence of *Escherichia coli* 26R 793, a Plasmid-Free Recipient Strain Commonly Used in Conjugation Assays

**DOI:** 10.1128/genomeA.00707-16

**Published:** 2016-10-20

**Authors:** Juan Wang, Daniel Hurley, Kathleen McGrath, Li Bai, Herbert Hächler, Roger Stephan, Séamus Fanning

**Affiliations:** a College of Veterinary Medicine, Northwest A&F University, Shaanxi, China; b UCD Centre for Food Safety, School of Public Health, Physiotherapy and Sports Science, University College Dublin, Belfield, Dublin, Ireland; c Key Laboratory of Food Safety Risk Assessment, Ministry of Health, China National Centre for Food Safety Risk Assessment, Beijing, China; d Institute for Food Safety and Hygiene, Vetsuisse Faculty, University of Zurich, Zürich, Switzerland; e Institute for Global Food Security, School of Biological Sciences, Queen’s University Belfast, Belfast, Northern Ireland

## Abstract

Here, we report the draft genome sequence of the lactose-negative, rifampin-resistant, *Escherichia coli* strain 26R 793. This isolate has been widely used in conjugation experiments as a general recipient strain.

## GENOME ANNOUNCEMENT

Since the isolation of the original *Escherichia coli* strain K-12 which was cultured from a feces sample taken from a diphtheria patient in 1922, a variety of mutant derivatives of *E. coli* K-12 have been generated for various laboratory assays (1). The lactose-negative, rifampin-resistant strain *E. coli* 26R 793 was one such derivative (2, 3). This strain has been used for over 20 years as a recipient for conjugation experiments with *E. coli*, *Klebsiella*, and *Salmonella* species (4–6).

In this study, we determined the draft genome sequence of *E. coli* 26R 793. Genomic DNA was purified from an overnight culture using the UltraClean Microbial DNA isolation kit (MO BIO Laboratories, Inc., USA) according to the manufacturer’s instructions. This template DNA was sequenced using the Illumina HiSeq 2500 platform (Illumina, San Diego, CA, USA) generating 100 bp paired-end reads from a library with an average insert size of 500 bp and a total amount of quality-filtered raw sequence of more than 600 Mbp. Reads were assembled *de novo* using SPAdes v3.6.2 (7) resulting in 102 contigs of over 200 bp. Annotation of these contigs was performed using RAST (8).

The *E. coli* 26R 793 genome was found to be 4.51 Mbp with a G+C content of 50.8%. It contained 4,335 predicted protein-coding sequences (CDS), five copies of rRNA operons, and 72 tRNA genes. An *in silico* multilocus sequence type (MLST) analysis was performed using the MLST targets. *E. coli* 26R 793 was assigned to sequence type (ST) 10 and the serotype was identified as H48.

When compared to the *E. coli* K-12 substrain MG1655 genome (GenBank accession number NC_000913.3), 42 genes of *E. coli* 26R 793 contained single nucleotide polymorphisms (SNPs) including seven synonymous and 19 non-synonymous mutations. Further, *E. coli* 26R 793 contained eight insertional mutations, six deletional mutations, and four indels located in the region of CDS. In addition to its *rpoB* gene variant (a single amino acid substitution at position 513, when compared with *E. coli* K-12 substrain MG1655) encoding resistance to rifampin, *E. coli* 26R 793 also possessed an enzymatic and inactivating *tet*(34) gene, and two virulence genes (*gad* and *iss*).

### Accession number(s).

The complete genome sequence of *E. coli* 26R 793 has been deposited at EMBL/GenBank under the accession number FLKR02000000.
